# ASO Author Reflections: Severe Morbidity After Major Surgery in Patients with MEN1

**DOI:** 10.1245/s10434-020-09540-0

**Published:** 2021-01-27

**Authors:** Dirk-Jan van Beek, Wessel M. C. M. Vorselaars, Inne H. M. Borel Rinkes, Menno R. Vriens

**Affiliations:** Department of Endocrine Surgical Oncology, University Medical Center Utrecht, Utrecht, The Netherlands

## Past

Formerly, extensive procedures such as the Thompson procedure (i.e., distal pancreatectomy to the level of the superior mesenteric vein, enucleation of tumors in the pancreatic head, duodenotomy with local excision of tumors in the duodenum, and peripancreatic lymph node dissection) were proposed to achieve cure for multifocal duodenopancreatic neuroendocrine tumors (dpNETs) in patients with multiple endocrine neoplasia type 1 (MEN1).^1^ A shift toward more conservative surgical indications as well as better localization enabling focused resections has taken place. Unfortunately, limited resections will not suffice. If major duodenopancreatic surgery is required, the pros and cons should be carefully weighted, but data on complications after major duodenopancreatic surgery in MEN1 are limited, and no studies have assessed the cumulative burden of complications.

## Present

The current study observed a severe complication (Clavien–Dindo grade ≥ 3) in nearly two-thirds of patients.^2^ Of those with severe morbidity, 76% had at least one more severe complication. Overall, the cumulative burden of complications was substantial. After pancreatoduodenectomy, patients had higher cumulative morbidity than after total pancreatectomy. Nevertheless, in the pancreatoduodenectomy group, one-third of patients did not suffer from any form of pancreatic insufficiency. The occurrence of complications could not be predicted by preoperative characteristics, which thus hampers preoperative decision-making. These results demonstrate the significant burden associated with major duodenopancreatic surgery in MEN1.

## Future

To improve patient care, future perspectives include optimization of equivocal surgical indications to facilitate improved preoperative patient selection as well as a reduction of the burden of complications once major surgery is performed.

First, more specific and mainly tumor-based risk factors are needed to improve patient selection and to abandon the “one size fits all” treatment principle; For example, recent clinical data imply that a shift from 2 to 3 cm as surgical cutoff might yield similar oncological outcomes for pNETs, and one might therefore consider a prolonged wait-and-scan policy in some patients.^3^ Transcription factors are currently being investigated for the purpose of tumor-based risk stratification.^4^ However, as these transcription factors depend on pre- or intraoperatively obtained tumor tissue, other techniques such as imaging-based risk stratification will likely be more patient-friendly and suit the radiological screening program in MEN1. Upcoming molecular imaging, particularly receptor-based imaging techniques, will hopefully enable imaging-based risk stratification. In the best-case scenario, these techniques will identify malignant dpNETs when these are still small and potentially eligible for enucleations because of size and favorable localization.^5^ In addition, further research is warranted to elucidate patterns of metastases since all patients undergoing pancreatoduodenectomy plus distal pancreatectomy suffered from a severe complication, which stresses that the oncological benefits should outweigh the complications associated with this procedure.

Second, centralization of care will likely improve patient outcomes in MEN1. Preoperative multidisciplinary and ideally multicenter team discussions will enable decision-making regarding the most complex patients with MEN1 within expert teams. When major surgery is considered, centralization of surgery within “high-volume” teams consisting of endocrine and hepatopancreatobiliary surgeons with vast experience in MEN1-related dpNETs should not be influenced by geography or travel distances and should potentially not be limited by national borders. Aims of these teams should be to reduce the incidence as well as the impact of complications. Whether patients with MEN1-related dpNETs have an increased risk of complications as compared with patients with sporadic dpNETs should be investigated in further studies to optimize referral patterns.
